# Microfluidic Rheology: An Innovative Method for Viscosity Measurement of Gels and Various Pharmaceuticals

**DOI:** 10.3390/gels10070464

**Published:** 2024-07-16

**Authors:** Zsófia Vilimi, Zsófia Edit Pápay, Bálint Basa, Xeniya Orekhova, Nikolett Kállai-Szabó, István Antal

**Affiliations:** Department of Pharmaceutics, Semmelweis University, Hőgyes E. Street 7-9, 1092 Budapest, Hungary; vilimi.zsofia@semmelweis.hu (Z.V.); papay.zsofia@semmelweis.hu (Z.E.P.); basa.balint@semmelweis.hu (B.B.);

**Keywords:** microfluidics, viscosity measurements, hydrogel, dosage forms, oral gel, vaginal gel, topical gel, carbomer gel, cellulose gel, carrageenan gel

## Abstract

Measuring the viscosity of pharmaceutical dosage forms is a crucial process. Viscosity provides information about the stability of the composition, the release rate of the drug, bioavailability, and, in the case of injectable drug formulations, even the force required for injection. However, measuring viscosity is a complex task with numerous challenges, especially for non-Newtonian materials, which include most pharmaceutical formulations, such as gels. Selecting the appropriate shear rate is critical. Since viscosity in many systems is highly temperature-dependent, stable temperature control is necessary during the measurement. Using microfluidics technology, it is now possible to perform rheological characterization and conduct fast and accurate measurements. Small sample volumes (even below 500 µL) are required, and viscosity determination can be carried out over a wide range of shear rates. Nevertheless, the pharmaceutical application of viscometers operating on the principle of microfluidics is not yet widespread. In our work, we compare the results of measurements taken with a microfluidic chip-based viscometer on different pharmaceutical forms (gels, solution) with those obtained using a traditional rotational viscometer, evaluating the relative advantages and disadvantages of the different methods. The microfluidics-based method enables time- and sample-efficient viscosity analysis of the examined pharmaceutical forms.

## 1. Introduction

Knowledge of the rheology of pharmaceutical dosage forms is extremely important during drug development, as it provides essential information about the stability of the dosage form, the release rate of the active pharmaceutical ingredient (API), bioavailability, and applicability [1,2]. In the case of injectable drug delivery systems (such as in situ forming gels as implants and injections), viscosity determines the force required for injection, which significantly affects the application process [3,4]. Under the influence of force, the behavior of liquids can vary greatly from each other. For Newtonian fluids, the relationship between shear stress and shear rate is linear, meaning that viscosity remains constant at any shear rate. For non-Newtonian fluids, the viscosity can change with the shear rates. This change can manifest as shear thickening or shear thinning, or the exhibition of mixed behavior across different shear rate ranges [5,6,7]. Furthermore, materials can exhibit viscoelastic behavior, possessing the ability to switch from a viscous to an elastic character and back [8]. Measuring viscosity can present numerous challenges, especially for non-Newtonian materials, which include most pharmaceutical formulations (gels, creams, etc.). Additionally, viscosity in many systems (including nano- and microsized preparations and gels) is highly temperature-dependent, necessitating precise temperature control during measurements [9]. Traditional viscosimeters may require large sample volumes, which can be impractical and expensive [10]. Among these traditional viscosimeters are rotational viscosimeters, which measure the torque required to rotate the geometry in the sample. They can be used to measure the viscosity of less viscous materials (e.g., mucilage, some gels) and more viscous materials (e.g., gels, creams, ointments), but only in a low shear range [11]. Oscillatory viscosimeters deform the sample using an oscillatory motion and measure the viscosity and elasticity from the sample’s response. They can provide more accurate results at lower shear rates for higher-viscosity samples, but despite this, both the viscosity and shear measurement ranges are wide [12]. Capillary viscosimeters measure the flow of a liquid under pressure through a narrow capillary and calculate the viscosity from this flow. They are suitable for measuring low-viscosity samples (solution, some gels) across a very wide shear range, even up to 10^6^ s^−1^ [13,14].

**Figure 1:**
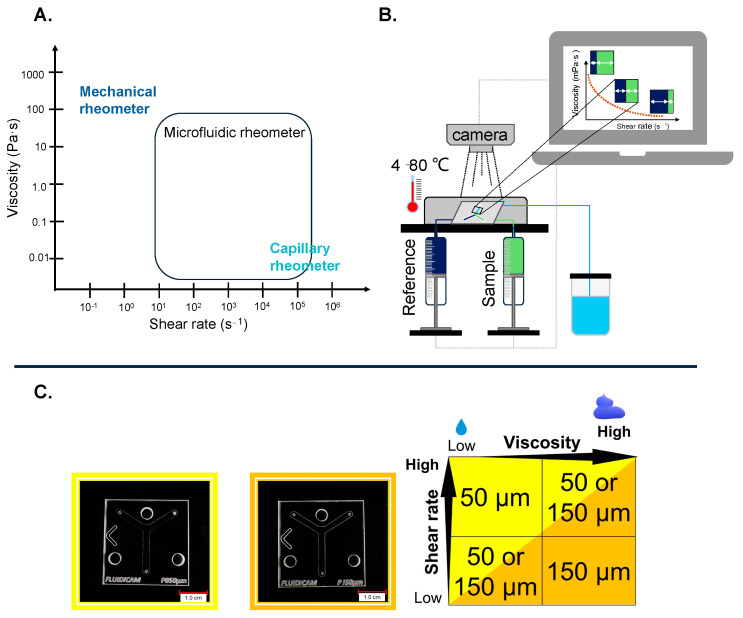
(**A**) Possibilities for determining viscosity. (**B**) Schematic drawing of the Fluidicam^TM^ RHEO. (**C**) Digital images (KEYENCE VHX 970) of plastic-based microfluidic device with Y-shaped channels 50 and 150 µm in diameter and their use under measurement conditions.

The wide range of fluid behaviors and shear conditions has necessitated the development of new experimental devices and methods for studying rheological properties. These devices can be grouped into macro- and microscale rheometers [15]. The most used are the previously mentioned macroscale rotational rheometers. However, studying the rheological behavior of some complex fluids with macrorheometry can be a complex and difficult task due to the technical limitations, which can significantly reduce the accuracy of the results. Other disadvantages of macrorheometry include the use of large sample volumes and the potential for liquid evaporation during the measurement process [1,16]. The latest developments in microfluidic technology offer promising solutions to these challenges [17]. Fluidicam^TM^ RHEO (Figure 1B,C) is a revolutionary device in the field of microfluidics-based viscosity measurements. The device combines microfluidic principles with optical detection, allowing for the rapid and precise determination of fluid viscosity during flow. The device is widely applicable not only in the pharmaceutical industry but also in the food, cosmetics, biotechnology, and material sciences [18]. The operation of the microfluidic viscosimeter is based on two syringes positioned before the microfluidic chip. These syringes are capable of generating mechanical displacement ranging from 0.5 µm/min to 30.0 mm/min, corresponding to flow rates from 1 µL/min to 4900 µL/min. One syringe contains the test sample, while the other holds the reference solution. The viscosity of the reference solution must be known very precisely at different temperatures to ensure accurate measurement evaluation. Ready-to-use references with known viscosity are available, but they can also be prepared in a laboratory. The color of the reference sample must differ from the measured sample so that the camera can recognize them. If using a laboratory-made reference sample, its viscosity needs to be measured beforehand at various temperatures using another viscosimeter. The two syringes push the reference and the sample through a Y-shaped channel from opposite sides, with dimensions of 2.2 mm × 50 µm or 150 µm. Different-diameter chips are interchangeable depending on the physical properties of the sample (Figure 1C). In addition, both plastic and glass chips are available, which should also be selected based on the sample’s physical properties (e.g., for low-viscosity solutions, or some gels, the smaller microchip is needed; for more viscous preparations and for aqueous dispersions, the bigger one is needed; and for oil-containing preparations, the glass microchip should be used). In the chip with a 150 µm gap, a shear rate range of 100–20,000 s^−1^ can be achieved, while in the smaller chip with a 50 µm gap, a higher shear rate range of 1000–180,000 s^−1^ is attainable. Before the sample and the reference solution enter the microfluidic chip, they pass through a 100 µL heat exchanger chamber, which immediately ensures temperature control. Then, the two fluids enter the Y channel arms simultaneously and meet in laminar flow inside the channel. A high-resolution camera placed above the chip (capable of capturing 25–35 frames per second, depending on the computer’s performance) detects the surface positioning, which correlates with the viscosity of the fluids. This enables the plotting of the viscosity profile as a function of the fluid flows and speed [19].

Very small sample amounts are needed for each measurement. Additionally, the measurement process can be automated, with the instrument capable performing a specified number of parallel measurements without changing the syringe containing the sample. It can measure at different temperatures, allowing for the differentiation between the viscosity of a formulation intended for implantation stored in a potentially cool place and the same formulation’s viscosity at room and body temperatures. Its main advantage over traditional viscosimeters is time efficiency, reducing measurement times from hours to minutes. In our work, we aimed to compare the efficiency of a microfluidic chip-based viscosimeter and a traditional rotational viscosimeter in measuring the viscosity of various pharmaceutical forms. By evaluating the advantages and disadvantages of the two methods, we aim to highlight the potential of microfluidic methods for time- and sample-efficient viscosity analysis in drug development. Our results could contribute to the broader application of microfluidic technologies, thereby increasing the efficiency.

## 2. Results and Discussion

**Figure 2:**
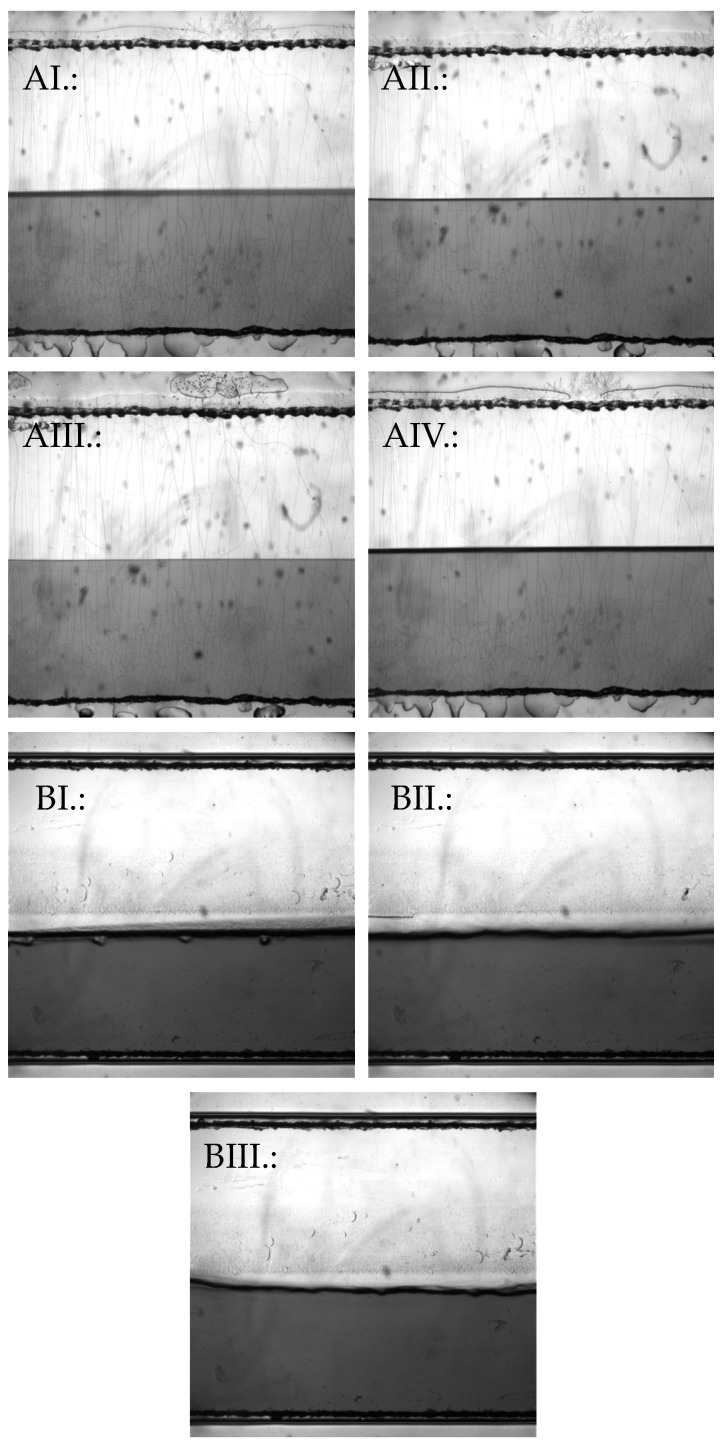
Comparison of the different measurements in the two plastic microfluidic chips. Liquid dosage forms in the smaller 50 µm microchip; (**AI**.:) Injection. (**AII**.:) Klysma. (**AIII**.:) Eyedrop. (**AIV**.:) Lubricant. Gel-based dosage forms in the bigger 150 µm microchip; (**BI**.:) Wound gel. (**BII**.:) Oral gel. (**BIII**.:) Vaginal gel.

In the following, we present the results of measurements of various solution- and gel- based pharmaceutical forms using the Kinexus Pro+ rotational rheometer and the Fluidicam^TM^ RHEO microfluidic viscosimeter, comparing them to each other and highlighting their relative advantages and disadvantages. The results also address the number of samples required for the measurements. The Kinexus Pro+ rheometer is a high-tech instrument for a wide range of rheological measurements, both in rotational and oscillatory modes. However, it provides reliable results for the examined pharmaceutical forms only within a limited shear range rate. The Fluidicam^TM^ RHEO is designed for a wide range of rheological measurements, particularly excelling in high-shear-rate applications, such as injecting, spraying, or 3D printing. While it offers precise and reliable results for various pharmaceutical formulations, its accuracy is most notable within specific, higher-shear ranges. The adjustable shear range depends on the selected microchip and the rheological properties of the sample. Figure 2 shows images taken during the measurement of the various pharmaceutical dosage forms.

### 2.1. Time and Sample Quantity

The table below (Table 1) shows that for identical samples, the measurement times are significantly shorter when using the microfluidic rheometer. The duration of measurements naturally depends on the width of the shear range for both instruments. The rotational rheometer uses a fixed sample quantity for each measurement, while for the microfluidic rheometer, this also depends on the shear settings. The time difference includes the fact that the microfluidic rheometer does not require sample change between parallel measurements. It is possible because the sample to be measured is preloaded into the syringe, allowing the next parallel measurement to start automatically after one is completed. In contrast with the rotational viscosimeter, the sample must be placed onto the sample holder plate, and after the measurement time has elapsed, it must be properly removed, and the sample holder plate must be thoroughly cleaned. It is understandable that the cleaning times vary based on the physical properties of the samples.

When evaluating the viscosity results, we took into consideration the possible shear ranges occurring at the site of use (vaginal environment, spreading on the skin, ingestion, etc.) as well as those occurring during application (injection, dispensing from the tube, etc.).

### 2.2. Parameters of the Applicators, Force Required for Extrusions

In order to predict the shear range occurring during application (e.g., rectal/vaginal applicators) or injection, for the tested formulations where this parameter is not negligible during use, we measured the force required to extrude the preparations from the applicator and the applicator parameters (length, hole size) (Table 2).

### 2.3. Liquid Dosage Forms

**Figure 3:**
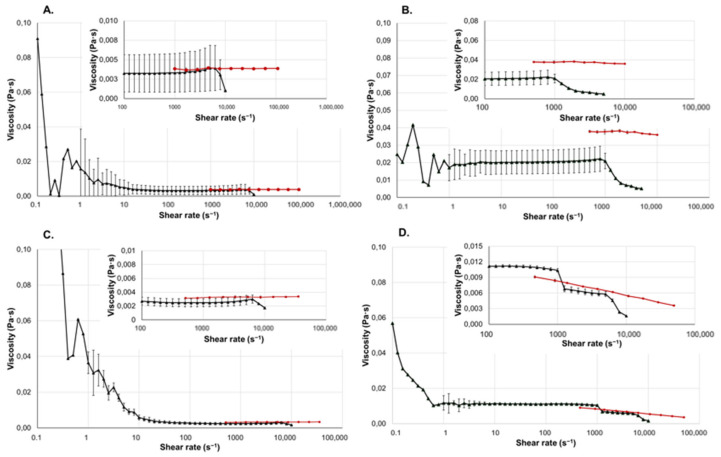
Viscosity curves of the liquid dosage forms. The black curve shows the measurement results obtained with the Kinexus Pro+, and the red curve shows the results obtained with the Fluidicam^TM^ Rheo. In the top right corner, an enlarged graph of certain ranges of the viscosity curves can be seen. All the investigated dosage forms were measured three times, and all parallels were measured from freshly-loaded samples. (**A**) Injection. (**B**) Lubricant. (**C**) Klysma. (**D**) Eyedrop.

The results of the viscosity measurements of the liquid pharmaceutical forms are shown in Figure 3.

The investigated eyedrop is an ophthalmic preparation mainly used for the treatment of various types of eye irritation. The product promotes the regeneration and protection of the eye’s mucous membrane and helps prevent bacterial and viral infections [20]. The product contains polysorbate 80, a nonionic surfactant, which can affect its viscosity [21]. Since this formulation has low viscosity, the rotational viscosimeter cannot provide accurate measurement results either in the low or high shear range. Between 1 and 1000 s^−1^, it exhibits Newtonian properties. However, blinking can create such a high shear rate range (4000 to 28,000 s^−1^) that the rotational viscosimeter can no longer provide reliable results. With the microfluidic viscosimeter, the rheological properties of the eyedrop are measurable on these high shear rates. The results show that the eyedrop also exhibits slight shear thinning behavior at higher shear rates, which is particularly important to know when formulating ophthalmic preparations.

The Klysma Diazepam Desitin is a solution containing diazepam as the active ingredient. It is indicated for the emergency treatment of epileptic seizures and is used to relieve severe muscle spasm due to its muscle relaxant effect [22]. Its rectal route of administration via enema is particularly advantageous when the patient is unable to take medication orally (e.g., during a seizure). Among its components, propylene glycol (PG) has the most significant impact on viscosity. PG is a synthetic liquid excipient, which absorbs water. Due to its safety, it is widely used in the food and pharmaceutical industries [23]. Based on the measurements of both instruments, the formulation exhibited Newtonian behavior in the shear range of 10 to 50,000 s^−1^. In the range below 10 s^−1^, the rotational viscosimeter does not provide reliable results. The shear rate within the rectum typically ranges between 10 to 100 s^−1^. By knowing the force required to extrude the formulation from the tube, it is possible to calculate the approximate shear rate that will occur during application [24].

The tested injection contains metamizole sodium, primarily used for fever and pain relief. Due to its rapid onset and effectiveness, it is particularly suitable for treatment of severe or acute pain and high fever when oral medications are inadequate or cannot be administered [25]. It does not contain any viscosity-modifying agents. The formulation showed Newtonian behavior when measured with both instruments. However, the measurement made with the rotational viscosimeter is not reliable under 10 s^−1^. The microfluidic viscosimeter, on the other hand, provided reliable results for our samples at on higher shear rates.

Implants are often equipped with lubricants and coatings, where appropriate viscosity is critical for the implant’s functionality. Polyethylene glycol 400 (Macrogola 400) is a low-molecular-weight grade of polyethylene glycol, widely used in pharmaceutical and medical applications due to its excellent solubility, low toxicity, and lubricant properties [26,27]. A microfluid-based rheometer can assist in the precise measurement and control of lubricants and coatings. Implants must be biocompatible with the human body to minimize the chances of unwanted immune responses and tissue reactions. Viscosity measurements can help understand the viscosity of fluids interacting with implants, thus facilitating biocompatibility studies [28,29].

In the shear rate ranges where the results were visibly unreliable, the deviations were not indicated. It can be observed that the deviation values of the microfluidic rheometer are negligible.

### 2.4. Gel-Based Dosage Forms

**Figure 4:**
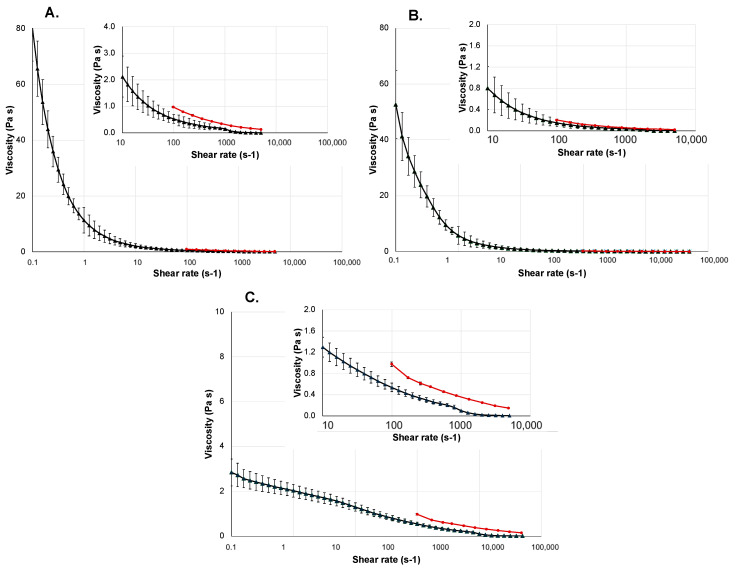
Viscosity curves of gel-based dosage forms. The black curve shows the measurement results obtained with the Kinexus Pro+, and the red curve shows the results obtained with the Fluidicam^TM^ Rheo. In the top right corner, an enlarged graph of certain ranges of the viscosity curves can be seen. All the investigated dosage forms were measured three times, all parallels were measured from freshly-loaded samples. (**A**) Wound gel. (**B**) Oral gel. (**C**) Vaginal gel.

The results of the viscosity measurements of the gel-based dosage forms are shown in Figure 4.

The tested vaginal gel is used for the treatment and prevention of vaginal bacteriosis and vaginal candidiasis. The gel product contains lactic acid and glycogen, which help reduce vaginal pH, thereby creating the pH value necessary for proliferation of bacteria exposed to vaginal microflora [30,31,32]. Its main thickening and gelling agent is methyl-hydroxy-propyl-cellulose (MHPC), which increases the viscosity of the preparation, making it a hydrogel. MHPC is a cellulose derivate widely used in the cosmetic and pharmaceutical industries to set the required consistency [33]. The vaginal gel is applied directly in the vagina using an applicator. The biologically relevant shear rate interval regarding the vaginal environment is 0.01 s^−1^ to 100 s^−1^ [34]. The viscosity measurement of Lactofeel vaginal gel with the rotational viscosimeter was conducted in the range between 0.1 and 5000 s^−1^. It is a low-viscosity hydrogel, with the stable measurement range between 1 and approximately 1000 s^−1^. This covers most of the shear range occurring in the vaginal environment, but during application, the small-diameter applicator induces higher shear rates. The viscosity results of the gel measured by the microfluidic rheometers and the rotational viscosimeter show very close correlations within the invested ranges; the difference between the viscosity measurements obtained from the two instruments is at most 0.5 Pa·s.

The wound-healing gel can be used for the treatment of minor wounds, cuts, abrasions, and burns. The main active ingredient of this gel is hyaluronic acid, which plays an important role in the wound-healing process and helps maintain the moisture content of the wound, resulting in faster healing [35]. Its gelling is carbomer, a typical excipient in hydrogels, a high-molecular-weight polymer of acrylic acid crosslinked with allyl ethers of polyalcohol. Gels made with this gelling agent are easy to apply on damaged skin, they spread well, and they form a thin film that cools down the skin [36]. The gel exhibits a highly pronounced shear thinning behavior. The two shear factors that may occur during use are the shear ranges on the skin during spreading and the shear range during dispensing from the tube, along with the required force for this. The shear rate during the spreading of the gel on the skin is approximately around 100 s^−1^ [37]. The measurement results show that the tested hydrogel formulation significantly thins, even with very small increases in shear rates. The viscosity results for the wound gel obtained with the microfluidic rheometer and the rotational viscosimeter are highly comparable, with differences between the measurements not exceeding 0.5 Pa·s at any measurement point.

Gloup is a viscous, gel-formed medical device that facilitates the ingestion and swallowing of medications by coating solid dosage forms [38]. It is a particularly significant oral gel in pediatrics and geriatrics, as well as for patients with swallowing difficulties. Its gelling agent is carrageenan, a naturally occurring polysaccharide capable of forming gel in water and a widely used food additive [39]. Shear rates during swallowing can vary between 10 and 50 s^−1^ [40]. The shear thinning behavior of the gel-based medical device is extremely advantageous, allowing it to effectively fulfill its purpose of easing swallowing. The results measured by the two methods are almost identical.

It can be seen that the deviation values of the microfluidic rheometer are almost negligible.

## 3. Conclusions

Based on our results, we can conclude that the different viscosity measurement methods have their own strengths and limitations depending on the application, the type of the formulation, and the aim of the measurement. The rotational viscosimeter provides reliable results over a wider shear range. This method proved effective for gel-like formulations (vaginal gel, wound gel, oral gel) that were conducted in the range of 0.1 to 5000 s^−1^. However, formulations with viscosities close to that of water did not yield evaluable results in the lower-shear-rate ranges, nor in the ranges occurring during injection or blinking. Another benefit of the rotational viscosimeter is that some samples can be partially recovered from the sample holder plate after the measurement.

Meanwhile, the microfluidic rheometer provided valuable insights for formulations experiencing higher shear rates during use or application. These formulations can be the injections, injectable gels, implants, eyedrops, or even ocular gels and the measured applicator-based pharmaceuticals (vaginal gel, Klysma). It is important to note that, since the investigated sample was mixed during the measurement process, microfluidic viscosimetry is a destructive measurement process, and the sample cannot be recovered post analysis.

In the ranges where the measurement capabilities of the microfluidic rheometer and the rotational viscosimeter overlapped, we obtained comparable results, although the microfluidic viscosimeter was able to measure with smaller deviations. The measurement process is quick, often taking only a few minutes. This efficiency is extremely beneficial, when multiple samples need to be analyzed in a short amount of time. Also, an advantage of the machine is that the system requires minimal sample preparation.

Overall, both methods are valuable tools for viscosity measurement, each with their specific applications and advantages. The microfluidic rheometer shows great potential for further developments, particularly in the analysis of formulations exposed to high shear rates, such as ophthalmic and injectable preparations.

## 4. Materials and Methods

### 4.1. Materials

In Table 3 below, the examined solution- and gel-based pharmaceuticals, all of which were purchased from a Hungarian pharmacy, are listed.

During the product selection, our goal was to study the impact of shear rate on viscosity across a wide range, from gel-type pharmaceutical forms with various viscosities to liquid formulations. An important aspect was measuring the effect of high shear rates on viscosity, such as the shear rate occurring during blinking for ophthalmic preparations or during injection for injectable drugs. We also aimed to determine whether a single measuring instrument could be used for comprehensive viscosity assessment of a pharmaceutical form, i.e., whether we can simulate with one device the shear range encountered at the point of use and the shear range occurring during application or administration.

### 4.2. Methods

Two different viscosimeters were used for the measurements, the Kinexus Pro+ rotational rheometer (Malvern Panalytical, Worcestershire, UK) and the Fluidicam^TM^ RHEO viscosimeter (Fromulaction, Toulouse, France). The settings were adjusted in each case to the specific characteristics, conditions of use, and route of administration of the chosen solution- or gel-based dosage forms.

#### 4.2.1. Viscosity Measurements with Kinexus Pro+

The rheological properties of the selected solution- or gel-based pharmaceutical formulations were first measured using the Kinexus Pro+ rotational rheometer (Malvern Panalytical, UK). This rotational rheometer system can apply controlled shear deformation on the tested samples, allowing the measurement of flow properties and dynamic material properties. The Kinexus Pro+ rheometer is designed to measure viscosities ranging from low-viscosity fluids to higher-viscosity substances. Its measurement range covers viscosities from 1 mPa·s to 10,000 Pa·s. The device can accurately measure shear rates from 10^−6^ to 10^4^ s^−1^; however, it is highly dependent on the physical characteristics of the tested samples [42]. Depending on the viscosity of the products, we used cone–plate (CP4/40 SR0207 SS: PL65 S0815 SS) and plate–plate (CP1/50 SR1222 SS: Solvent Trap 55 mm C0157 SS) geometries with the rheometer. All measurements were performed at a temperature regulated by a Peltier plate, appropriate to the route of administration. These temperatures are also listed in the table below (Table 4). The shear rate range was also selected either based on the shear ranges encountered during administration or based on the environment of application, as permitted by the device.

#### 4.2.2. Viscosity Measurements with Fluidicam^TM^ RHEO

The viscosities were measured using the Fluidicam^TM^ RHEO viscosimeter (Formulaction, France), which operates based on microfluidic principles. The shear rate and the temperature were selected according to the route of administration for each pharmaceutical formulation. Appropriate reference solutions to the viscosity of each pharmaceutical dosage forms (gels, solution, lubricant) were used. These liquid references (Formulaction, France) had different viscosities, with targets of 5, 50, and 500 mPa s at 25 °C. Table 5 presents the measuring settings.

**Figure 5:**
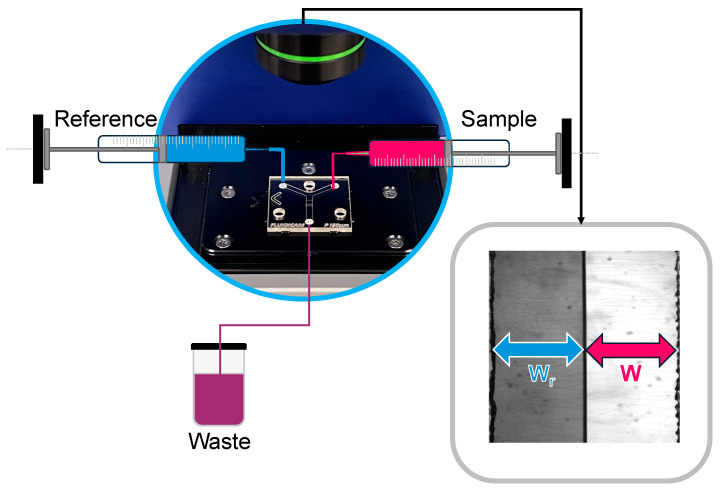
The principle of viscosity calculation with the Fluidicam^TM^ RHEO. Measurement of distilled water compared to a 5 mPa·s (25 °C) reference solution.

Based on its operation, the microfluidic rheometer is suitable for measuring biological samples, examining various liquid or even semisolid pharmaceutical forms (oral gels, wound gels, vaginal gels), lubricants for implants, implants themselves, and their surrounding environments. However, since it operates based on flow, the sample must be homogeneously fluid. Very dense, viscous materials can only be measured if they exhibit shear-thinning properties. Injectability is an important consideration in the design and development of parenteral dosage forms, such as implants. During injection, the formulation is subjected to very high shear forces. With the help of the microfluidic viscosimetry, it can be examined whether high shear forces positively facilitate injectability or negatively impact it. In the small-sized chips, laminar flow (Figure 5) is continuously ensured for both the reference and the sample, which makes it straightforward to calculate the sample’s viscosity in the following manner.

(1) $$\frac{W}{W_{r}}=\frac{\eta }{\eta_{r}}\cdot \frac{Q}{Q_{r}}$$

*W* and *W_r_*—the measured widths by the camera; η—the viscosity of the sample; $\eta_{r}$-the known viscosity of the reference; *Q* and *Q_r_*—the flow rates set by the equipment; subscripted r always refers to the reference solution. The flow rate is adjusted by the equipment based on the width of the reference and the tested samples within the microchip. The shear rate is defined as the quotient of the change in velocity and the width of the channel in the microchip [43]. With the previous data and knowledge of the reference solution’s viscosity, the viscosity of the tested sample can be easily calculated.

Examining ocular dosage form (eye drops, ocular gels, etc.) is particularly suitable with this method, because during blinking, the eye can exert high shear rates, which a rotational viscosimeter is not capable of measuring [44,45]. The Fluidicam^TM^ RHEO can imitate it and allows the determination of the formulation’s suitability for ophthalmic use. The shear force during different usage methods can depend on numerous factors. These include the injection force or the force of squeezing from a tube/applicator, the viscosity of the dosage form, the speed of injection or squeezing, the diameter of the needle or applicator, and the properties of the media, where we insert or inject the dosage form. Generally, the shear force can be determined as follows [46,47]:

(2) $$\tau =\eta \cdot \frac{du}{dy},$$

τ—shear stress (Pa); η—viscosity (Pa·s); $\frac{du}{dy}$ is the velocity gradient (s^−1^). The velocity gradient refers to the shear rate, which is the velocity difference between the layers of the fluid per unit distance. It can be described mathematically as follows:

(3) $$\gamma =\frac{du}{dy},$$

where γ—shear rate (s^−1^); $\frac{du}{dy}$—velocity gradient (s^−1^).

#### 4.2.3. Digital Caliper

For pharmaceutical dosage forms (vaginal gel, klysma), where the shear rate during application could affect usage, we determined the diameter and length of the applicators and nozzles using an Aerospace digital caliper (Kovea Co. Ltd.; Incheon, Republic of Korea; 0.01 ± 0.02 mm accuracy) [48]. Based on the data, we determined the potentially occurring shear ranges. Five parallels were measured on each tube.

#### 4.2.4. Extrudability

For the dosage forms, where significant shear occurs during use (vaginal gel, Klysma), the force required to extrude the product from the tube was measured with a Stokes Handheld Hardness tester (F. J. Stokes Machine Company, Philadelphia, U.S.; Scale: 0–20 kg) [49]. Three parallels were measured from each sample.

The shear rates occurring during the administration of injections, injectable preparations, or even preparations that can be used with an applicator (vaginal gel, Klysma) depend significantly on the resistance at the injection site, the viscosity of the parenteral preparation, and the diameter of the needle used, and in the case of applicator-based products on the place of administration, the diameter, and the length of the applicator, and the viscosity of the product. The diameter of the needle is defined by the so-called gauge number. Needles with higher gauge number, meaning smaller diameter, result in higher shear rates at the same flow rate. For Newtonian injections, and for some applicator-based preparations (like the chosen Klysma), the shear rate can be calculated as follows:

(4) $$\Delta P=\frac{F}{A},$$

where ΔP—pressure drop (Pa); F—the force required to extrude the preparation from a tube/applicator, or the force needed to press the syringe (N); *A*—the area of the nozzle/tube (m^2^). To find the volumetric flow rate, the Hagen–Poiseuille equation should be used:

(5) $$Q=\frac{\pi r^{4}\Delta P}{8\eta L},$$

Q—volumetric flow rate ($\frac{m^{3}}{s}$); r—radius of the nozzle (m); η—viscosity of the fluid (Pa·s); *L*—the length of the pipe. Based on the results of Equations (4) and (5), knowing the volumetric flow rate and the pressure drop and the radius of the used nozzle, the shear rate can be approximated as

(6) $$\gamma =\frac{4Q}{\pi r^{3}}$$

## Figures and Tables

**Table 1:** Solution- and gel-based samples’ quantity and duration required for measurements with Fluidicam^TM^ RHEO and Kinexus Pro+.

Solution and Gel-Based Product	Used Sample/Measurement (mL)		Time/Measurement (min)	
	Kinexus Pro+	Fluidicam^TM^ RHEO	Kinexus Pro+	Fluidicam^TM^ RHEO
Vaginal gel	1.50	2.1	31 min ± 2 min	4 min 58 s ± 27 s
Wound gel	1.50	2.6	27 min ± 2 min	5 min 32 s ± 29 s
Eyedrop	1.19	1.2	48 min ± 4 min	3 min 12 s ± 12 s
Klysma	1.19	0.9	35 min ± 2 min	2 min 53 s ± 21 s
Injection	1.19	1.0	38 min ± 3 min	2 min 48 s ± 17 s
Lubricant	1.19	1.8	30 min ± 2 min	3 min 8 s ± 24 s
Oral gel	1.50	2.16	32 min ± 2 min	5 min 16 s ± 28 s

**Table 2:** Applicator parameters.

Solution- and Gel-Based Product	Diameter (mm)	Length (mm)	Force (N)
Vaginal gel	4.02	44.49	49.05
Klysma	1.91	44.59	49.05
Injection	Gauge	Gauge	-

**Table 3:** Commercially available, tested solution- and gel-based products with some information [20,22,25,30,35,38,41].

Dosage Form	Route of Administration	API	Viscosity Modifying Excipient	Indication	Brand Name	Manufacturer
Gel	Vaginal	Acidum lacticum, Glicogen	Methyl-hydroxy-propyl-cellulose (MHPC)	Bacterial vaginosis, Candidiasis	Lactofeel	Exeltis, Florham Park, NJ, USA
Gel	Topical	Sodium hyaluronate	Carbomer	Wound care	Curiosa	Richter Gedeon Nyrt., Budapest, Hungary
Solution	Eyedrop	-	Polysorbate 80	Lubricant	Phyteneo Occusept	Neofyt spol. s r.o., Stříbrná Skalice, Czech Republic
Klysma (solution)	Rectal	Diazepam	Propylenglycol	Seizure resolution	Diazepam Desitin 10 mg	Desitin Arzneimittel GmbH., Hamburg, Germany
Injection	Intramuscular/intravenous	Metamizole-sodium	-	Painkiller	Algopyrin 1 g/2 mL injection	Sanofi-Aventis, Paris, France
Excipient	Subcutaneous	-	-	Lubricant for implants	Macrogola 400	MAGILAB Kft., Budapest, Hungary
medical device-gel	Oral	-	Carragenan	Lubricant for solid dosage forms	Gloup	M. Technologies Fr., Tourcoing, France

**Table 4:** Kinexus Pro+ setting parameters.

Product	Geometry	Shear Rate Range (s^−1^)	Temperature (°C)
Vaginal gel	Cone–Plate	10^−1^ to 1 × 10^4^	37
Wound gel	Cone–Plate	10^−1^ to 5 × 10^3^	32
Eyedrop	Plate–Plate	10^−1^ to 1 × 10^4^	34
Klysma	Plate–Plate	10^−1^ to 5 × 10^3^	37
Injection	Plate–Plate	10^−1^ to 1 × 10^4^	37
Lubricant	Cone–Plate	10^−1^ to 5 × 10^3^	37
Oral gel	Cone–Plate	10^−1^ to 5 × 10^3^	37

**Table 5:** Fluidicam^TM^ RHEO setting parameters.

Product	Diameter of Gap Microchip (µm)	Shear Rate Range (s^−1^)	Temperature (°C)	Viscosity of Ready-to-Use Reference (mPa·s)
Vaginal gel	150	1 × 10^2^ to 5 × 10^3^	37	500
Wound gel	150	1 × 10^2^ to 5 × 10^3^	32	500
Eyedrop	50	5 × 10^2^ to 1 × 10^4^	34	5
Klysma	50	5 × 10^2^ to 3.5 × 10^4^	37	5
Injection	50	1 × 10^3^ to 1 × 10^5^	37	5
Lubricant	50	5 × 10^2^ to 1 × 10^4^	37	5
Oral gel	150	1 × 10^2^ to 5 × 10^3^	37	500

## Data Availability

The data presented in this study are openly available in article.
